# Comprehensive Assessment of Reference Gene Expression within the Whitefly *Dialeurodes citri* Using RT-qPCR

**DOI:** 10.3390/genes15030318

**Published:** 2024-02-28

**Authors:** Weizhen Kong, Xiaolu Lv, Xiaotong Ran, Marguerite Mukangango, Bugenimana Eric Derrick, Baoli Qiu, Changfei Guo

**Affiliations:** 1 Engineering Research Center of Biological Control, Ministry of Education, South China Agricultural University, Guangzhou 510640, China; kwz17866928844@163.com (W.K.); lxl17803903494@163.com (X.L.); rxt283629@stu.scau.edu.cn (X.R.); 2 Engineering Research Center of Biotechnology for Active Substances, Ministry of Education, Chongqing Normal University, Chongqing 401331, China; 3 College of Agriculture, Animal Sciences and Veterinary Medicine, University of Rwanda, Musanze P.O. Box 210, Rwanda; mukangango2@gmail.com (M.M.); isimbiella@gmail.com (B.E.D.)

**Keywords:** *Dialeurodes citri*, reference gene, RT-qPCR analysis, gene expression, experimental conditions

## Abstract

The citrus whitefly, *Dialeurodes citri*, is a destructive pest that infests citrus plants. It is a major vector in transmitting plant viruses such as citrus yellow vein clearing virus (CYVCV), which has caused severe economic losses worldwide, and therefore efficient control of this pest is economically important. However, the scope of genetic studies primarily focused on *D. citri* is restricted, something that has potentially limited further study of efficient control options. To explore the functionalities of *D. citri* target genes, screening for specific reference genes using RT-qPCR under different experimental conditions is essential for the furtherance of biological studies concerning *D. citri*. The eight candidate reference genes were evaluated by dedicated algorithms (*geNorm*, *Normfinder*, *BestKeeper* and Δ*Ct* method) under five specific experimental conditions (developmental stage, sex, tissue, population and temperature). In addition, the *RefFinder* software, a comprehensive evaluation platform integrating all of the above algorithms, ranked the expression stability of eight candidate reference genes. The results showed that the best reference genes under different experimental settings were *V-ATP-A* and *RPS18* at different developmental stages; *α-tubulin, 18S* and *V-ATP-A* in both sexes; *EF1A* and *α-tubulin* in different tissues; *Actin* and *Argk* under different populations; and *RPS18* and *RPL13* in different temperatures. The validation of selected reference genes was further identified using heat shock protein (*Hsp*) 70 as a reporter gene. Our study, for the first time, provides a detailed compilation of internal reference genes for *D. citri* that are suitable for RT-qPCR analysis, which is robust groundwork for comprehensive investigation of the functional target genes of *D. citri.*

## 1. Introduction

The citrus whitefly, *Dialeurodes citri* (Ashmead) (Heteroptera: Aleyrodidae)*,* is one of the most destructive whitefly species worldwide. It has caused serious economic losses in China [1,2]. In addition to citrus, *D. citri* can also harm deciduous plants from 30 different families, such as peach, persimmon and chestnut [3]. *D. citri* prefers a shaded environment, and damage therefore is often more serious in poorly ventilated and light-permeable orange orchards. *D. citri* adults migrate easily on wind currents and spread quickly, and the bodies of immatures are protected by wax, thus making them more resistant to pesticides. Nymphal stages usually fixate on the abaxial surface of newborn shoots, where they feed by ingesting plant sap and then consequently excrete a large amount of honeydew, which induces coal stain disease and, in severe cases, affects the quality and yield of the fruits [3,4]. In addition, *D. citri* can transmit many plant viruses, such as citrus yellow vein clearing virus (CYVCV), which has caused serious economic losses worldwide [4,5]. Currently, the control of *D. citri* largely remains dependent on chemical pesticides; however, the extensive use of chemical insecticides has produced serious negative impacts, especially insecticide resistance [6]. Given that efficient control of *D. citri* is of great economic importance worldwide, novel and sustainable alternative technologies need to be further explored.

Gene expression analysis has become a key research tool in various fields of life sciences [7,8,9,10,11]. Such in-depth studies provide a way to explore genes related to agricultural and forestry pests, aid in resolving the complex network of gene expression regulation and provide a solid scientific basis for effective agricultural and forestry pest control [12,13,14]. Gene expression quantification methods on the transcriptional level, like real-time quantitative PCR (RT-qPCR), ribonuclease protection assay (RPA) and Northern blotting, as well as quantification methods at the protein level, such as Western blot, require the use of reference genes to calibrate the expression of target genes and to correct the expression of the target gene to ensure that the results are accurate and reliable [15,16,17].

Housekeeping genes are also known as internal reference genes. Ideally, they can be stably expressed under the conditions of various experimental factors [11]. There are hundreds of housekeeping genes, with the most frequently used ones being *Actin, GAPDH, Tubulin, 18SrRNA* and *28SrRNA* [9,13,14,15,17,18,19,20,21,22,23,24,25,26,27,28,29]. However, numerous studies have demonstrated that the so-called constant expression of any one of these housekeeping genes is only a “range” of constant expression in a certain type of cell or under the effect of an experimental factor, while in other types of cells or under experimental conditions, there are variations, which can sometimes be a dozen-fold, tens of folds or even hundreds of folds of differences [12,16,18,19,20,21,25,26,27,30,31]. In addition, there is no absolute generalization of the internal reference genes; if they are used directly without screening, they may lead to wrong or even opposite conclusions concerning the target gene [12,14]. Therefore, the validation and screening of internal reference genes [8,25,32] should be carried out before gene expression analysis in order to select a more stable internal reference gene. This then determines the suitable internal reference gene for RT-qPCR and for the correction of the expression of the target gene in order to obtain true and reliable results [12,13,16].

Real-time fluorescence quantitative PCR, a powerful method for detecting gene expression profiles [8,33,34], is not only used for determining gene expression patterns but is also widely used to analyze differences in gene expression under different conditions, such as different time periods or samples that have undergone different treatments. This technique is also commonly used for verifying the accuracy and reliability of high-throughput sequencing (RNA-Seq) data of the transcriptome [11,17,18,19,34,35].

Due to the lack of information on the internal reference genes of *D. citri*, associated biological and functional genomics and proteomics studies have been largely limited. The correct selection of internal reference genes is crucial to ensure the accuracy of RT-qPCR results [12,14]. To this end, we screened eight commonly used reference genes from the transcriptome (SRA, No. SRP065471) of *D. citri*, including *Actin* (β-Actin), *RPL13* (ribosomal protein L13), *α-tubulin*, *18S* (18S ribosomal RNA), *V-ATP-A*, *RPS18* (ribosomal protein S), *EF1A* (elongation factor 1 *α*) and *ArgK* (arginine kinase) [17,18,19,20,27,28,29,30,31,32,35,36,37,38,39,40,41,42,43,44,45,46,47,48]. *V-ATP-A* and α-*tubulin* have proved to be the most optimal reference genes for different developmental stages and tissues in studies on *Coleomegilla maculata*, and studies on *Diaphorina citri* have demonstrated that *V-ATP-A* is expressed very stably at all developmental stages [17,19]; in a *Henosepilachna vigintioctopunctata* study, *RPS18* was regarded as the best reference gene for different temperatures [22]; and *ArgK* showed the highest applicability as a reference gene in *Lysiphlebia japonica* and *Spodoptera litura* [18,27,32,36]. In the *Sesamia inferens* endogenous gene screen, *18S* was shown to be optimal for different developmental stages and for sex [37]. *EF1A* is the most reliable reference gene for different tissues of *Plutella xylostella* and *Podoptera exigua* [38]. *RefFinder* software was utilized to synthesize four evaluation programs *(geNorm*, ∆*Ct* method, *NormFinder* and *BestKeeper)* to evaluate the expression stability of these genes under five experimental environment settings. To further validate our screening results, we used heat shock protein 70 (*Hsp70*) as a reporter gene [7,8,18,20,38]. The results of this study will not only accelerate the in-depth study of *D. citri* genomics but also provide a solid foundation for practical applications within the continual development of citrus whitefly control options.

## 2. Materials and Methods

### 2.1. Insect Rearing

The original citrus whitefly, *D. citri*, population used in this study was collected in May 2022 from the citrus orchard at the Institute of Fruit Research, Zhaoqing University, Guangdong China. The laboratory population was then reared in the glasshouse of the Engineering Research Center of Biological Control, Ministry of Education, South China Agricultural University (SCAU), Guangzhou China, using orange seedlings, under the following settings: temperature of 25 ± 3 °C, relative humidity of 60–90% and a photoperiod of 14 h:10 h (L:D) (the field population was first reared for five generations under laboratory conditions and then reared as a laboratory population).

### 2.2. Samples of Citrus Whitefly

The reference genes of *D. citri* were evaluated according to developmental stage, sex, body tissues of the adult, different populations and different temperature treatments of *D. citri*. The number of samples collected from *D. citri* at different developmental stages included 150 eggs, 80 young instar nymphs (1st/2nd instar), 50 older instar nymphs (3rd and 4th instar), 50 pseudopupae and finally 40 adults (20 adults of each sex); for sex-specific sampling, 20 females and 20 males at first-fledgling stage were taken. For the sampling of different body tissues, 80 first-fledged adults (40 female and 40 males) were dissected in phosphate-buffered saline (PBS) to obtain the head, thorax and abdomen in TRIzol. The different population treatments were categorized into laboratory and orchard populations, with 40 *D. citri* adults being collected from each population, respectively. In the temperature treatments, 100 first-fledged *D. citri* adults were introduced into nylon bags that covered young shoots of orange plantings and placed within an individual insect chamber at either 5 °C, 10 °C, 15 °C, 25 °C or 35 °C, respectively. Fifty *D. citri* samples (mixture of male and female) were then collected after 2 h from each temperature treatment for assessment.

A total of 50 µL of TRIzol Reagent (Invitrogen, Carlsbad, CA, USA) was added to the centrifuge tube, and the collected sample was placed into the tube for RNA extraction. Three times were set up for each experimental treatment.

### 2.3. Total RNA Extraction and cDNA Synthesis

RNA was extracted separately from the samples, using TRIzol reagent, following the test protocol outlined by Guo et al. [20]. RNA concentration was measured using a NanoDrop One spectrophotometer (Thermo Fisher Scientific, Waltham, MA, USA). Subsequently, the extracted RNA was dissolved in 30 µL of ddH_2_O. First-strand cDNAs from 1 µg of RNA from different samples were then synthesized using the PrimeScript RT Kit (Takara, Kyoto, Japan) according to the manufacturer’s protocol. The cDNAs synthesized were diluted 10-fold and used in the RT-qPCR reaction [13,18]

### 2.4. Gene Cloning and Primer Design

Eight candidate reference genes (*Actin*, α-*tubulin*, *EF1A*, *18S*, *RPS18*, *ArgK*, *RPL13* and *V-ATP-A*) were examined. These genes are commonly utilized as reference genes in RT-qPCR investigations involving insects. Primer pairs specific to *D. citri* were designed using Premier 5 software (v5.0) (refer to Table 1). The PCR reaction mixture (25 µL) included LA Taq DNA polymerase (Takara, Japan), and the PCR program followed the protocol described in Guo et al. [18,19,20]. Electrophoresis was run in TAE buffer (Beyotime, Shanghai, China) at 120 V for 20 min. The amplified PCR products were ligated into a pClone007 Blunt vector (TSINGKE, Beijing, China) and subsequently sequenced by BGI Company (Shenzhen, China) [18,20]. Finally, in order to gain insight into the function of the candidate reference genes and to simultaneously validate their applicability to gene expression studies, we scrutinized and analyzed the sequences of eight candidate reference genes, using the NCBI repository.

### 2.5. RT-qPCR of Each Reference Gene

To make a 50 µL reaction mixture, the following reagents were mixed: primers (5 µL) (including 2.5 µL of forward primer and 2.5 µL of reverse primer, both at 10 µM), SYBR Green Premix (Takara, Japan) (25 µL), diluted cDNA template (2.5 µL) and RNase-free water (17.5 µL) [18,20]. The mixture was divided into three technical replicates, each containing 15 µL of reaction mixture. All reactions were performed using the CFX96 Real-Time PCR System (Bio-Rad, Hercules, CA, USA). qPCR procedures consisted of an initial denaturation at 95 °C for 3 min, followed by 40 cycles of 95 °C for 10 s and 55 °C for 30 s. The qPCR procedure was performed using the CFX96 Real-Time PCR System (Bio-Rad, Hercules, CA, USA). In addition, a dissociation step cycle (55 °C for 10 s and then 0.5 °C for 10 s gradual increase in temperature up to 95 °C) was included for the dissociation curve analysis [18,20]. Slope analysis was performed by linear regression models, and RT-qPCR analysis was performed for each gene. Standard curves were generated for each primer pair based on serial dilutions of cDNA (5^−1^, 5^−2^, 5^−3^, 5^−4^ and 5^−5)^. The corresponding RT-qPCR efficiency (E) was calculated using the following formula: E = (10[−1/slope] − 1) × 100 [18,27].

### 2.6. Determination of Reference Gene Expression

Data from five experimental environment settings were analyzed separately. *RefFinder* (http://blooge.cn/RefFinder/) (accessed on 22 January 2024) combines four computational algorithms, namely *geNorm* [14], *NormFinder* [39], *BestKeeper* [40], and the ∆*Ct* method [41], to assess the stability of each of the eight reference genes. *geNorm* was proposed by Vandesompele et al. in 2002 to evaluate the reliability of reference genes independently of the number of cDNAs added, an algorithm that is very robust [14]. *geNorm* determines the minimum number of genes required for normalization by calculating the V-value. Initially, two candidate reference genes (normalization factor NF and NFn+1) are included to calculate the normalization factor ratio. If the ratio < 0.15 threshold (Vn/Vn+1 ≤ 0.15), the first two candidate reference genes are sufficient for normalization. However, if the ratio > 0.15, the candidate reference genes are added sequentially until the ratio drops below 0.15. If the first gene with a V-value of 0.15 appears after V2/3, it indicates that the two reference genes are sufficient for reliable normalization under the given experimental conditions. Vn/Vn+1 ≤ 0.15 implies that the n reference genes can be regarded as the best genes for RT-qPCR analysis [14,26,39,40,41].

*NormFinder* software uses a similar computational principle to the *geNorm* algorithm to screen for reference genes with stable expression. It determines the expression stability value of the candidate reference gene. Genes exhibiting the lowest stability values are deemed to be the most robust genes under the given experimental conditions [27,39].

The *BestKeeper* algorithm calculates expression levels of the target genes, considering parameters such as the correlation coefficient (r), standard deviation (SD) and coefficient of variation (CV) between the genes. The reference gene with the highest stability is identified by comparing the magnitude of expression level values of the candidate reference genes [27,40].

The Δ*Ct* method evaluates the expression level via a pairwise comparison of all candidate reference genes, and genes with lower SD values are considered to be the most stably expressed genes. Finally, *RefFinder* calculates the overall ranking of candidate reference genes by integrating four statistical methods (*geNorm*, *NormFinder*, *BestKeeper* and Δ*Ct* method) [14,26,41].

### 2.7. Gene Expression Level Analysis Using Various Reference Genes

Heat shock proteins (*Hsp*) are widely documented in insects and are one of the most important indicators of heat stress tolerance in insects. Studies have shown that they may play a key role in protecting cells from damage [42,49,50]. In view of the significance of this gene, we evaluated the robustness of the chosen candidate reference genes by utilizing *Hsp70* of *D. citri* as a target gene. The forward primer sequence of *Hsp70* is 5′-GATTAGACGTTTCGCGTCG-3′, and the reverse primer sequence is 5′-GAACCACGCCGAGTTATGTT-3′. *Hsp70* expression levels in *D. citri* were determined at different temperatures and tissues via normalization, using the two most reliable genes and the two least reliable genes.

By using the 2*^−^*^∆∆*Ct*^ method, the relative expression of *Hsp70* in the organism was computed. To determine if there were significant differences in *Hsp70* expression levels in *D. citri* under different experimental condition settings, an analysis was undertaken, using one-way ANOVA, which was performed in SPSS software (v18.0) (SPSS Inc., Chicago, IL, USA).

## 3. Results

### 3.1. PCR Amplification and Performance of Candidate Reference Genes in D. citri

**Figure 1:**
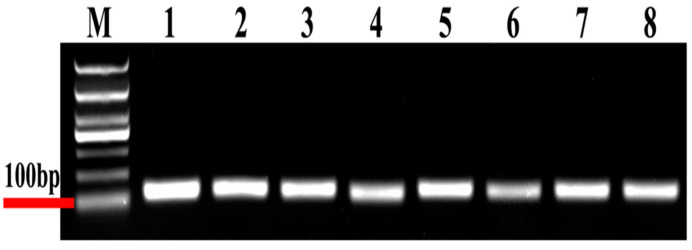
Electrophoresis of eight candidate reference genes. M, molecular marker. Templates in the PCRs are as follows (Lanes 1 to lane 8): (1) *18S*, (2) *V-ATP-A*, (3) *Actin*, (4) *ArgK*, (5) *α-tubulin*, (6) *EF1A*, (7) *RPL13* and (8) *RPS18.*

**Figure 2:**
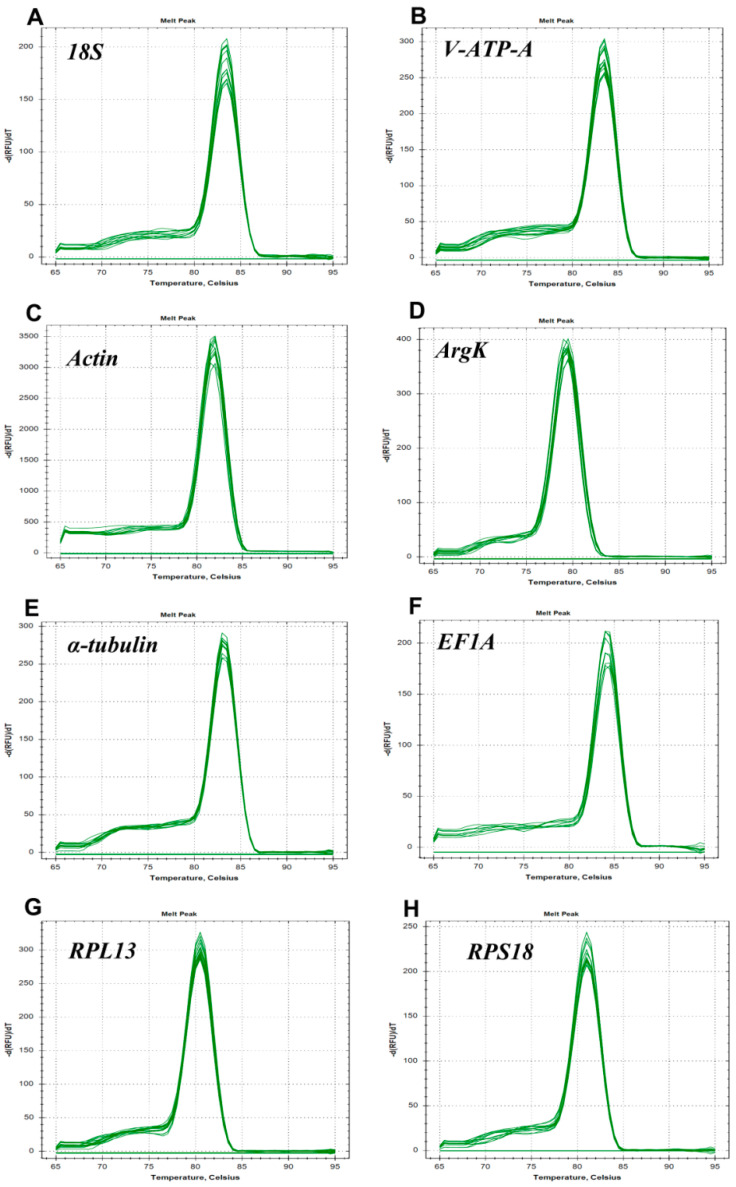
Melting curves of the eight candidate reference genes in *Dialeurodes citri*: (**A**) *18S*, (**B**) *V-ATP-A*, (**C**) *Actin*, (**D**) *ArgK*, (**E**) *α-tubulin*, (**F**) *EF1A*, (**G**) *RPL13* and (**H**) *RPS18.*

**Figure 3:**
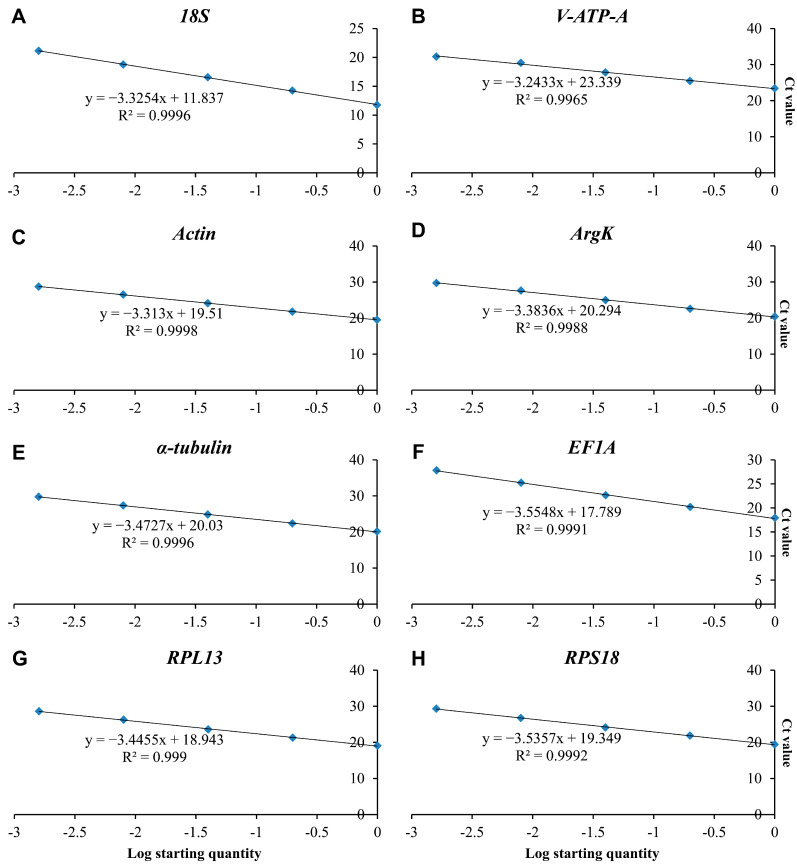
Standard curves of the eight candidate reference genes: (**A**) *18S*, (**B**) *V-ATP-A*, (**C**) *Actin*, (**D**) *ArgK*, (**E**) *α-tubulin*, (**F**) *EF1A*, (**G**) *RPL13* and (**H**) *RPS18.*

In the present study, eight candidate reference genes were expressed in *D. citri*, and a single band could be amplified via PCR for each gene, without non-specific PCR products (Figure 1). In melting curve analysis, all the reference genes were shown to be single peaks, thus confirming the specificity and accuracy of the primers (Figure 2). Table 1 lists the PCR primer amplification efficiencies (Es) and correlation coefficients (R2s) for the eight candidate internal reference genes, as well as the linear regression equations for primer amplification; all eight reference genes’ standard curves are shown in Figure 3.

### 3.2. Expression Levels of Reference Genes

**Figure 4:**
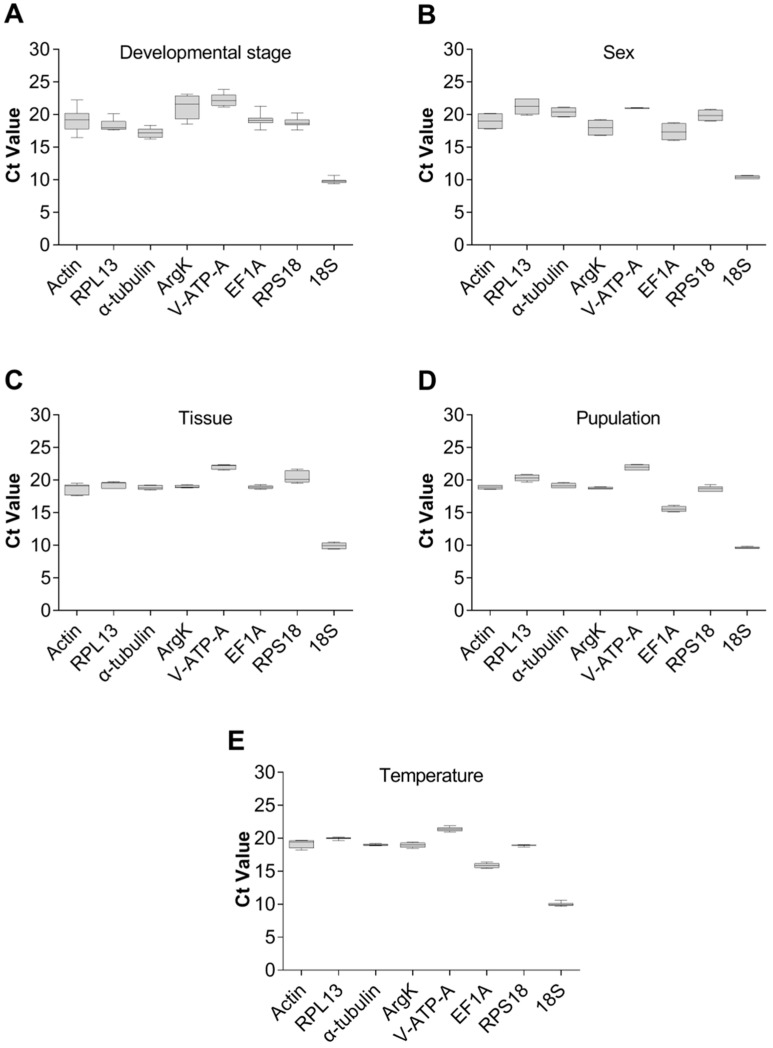
Expression of the eight candidate reference genes of *Dialeurodes citri* in five different experiments. The expression levels of the reference genes are shown in terms of the cycling threshold (Ct-value) for each experimental condition. (**A**) Developmental stage, (**B**) sex, (**C**) tissue, (**D**) population and (**E**) temperature.

The expression levels of eight candidate reference genes in five different experimental settings were assessed using the cyclic threshold (Ct) method. Under these conditions, the lowest Ct value for the eight candidate internal reference genes was 9.38, and the highest was 23.87 (Figure 4). Notably, *18S* had shown the lowest Ct value, indicating the highest expression level under the five experimental conditions, while the remaining candidate housekeeping genes had a minimum Ct value of 15.14 and a maximum of 23.87.

### 3.3. Stability of the Reference Genes under Specific Experimental Conditions

Under the five experimental conditions set, the stability of the eight candidate reference genes was assessed using the ∆*Ct* methods, *BestKeeper*, *NormFinder* and *geNorm* (Table 2). The lower the threshold value, the more robust the expression of the candidate reference gene. Typically, a threshold of 1.5 was used for software evaluation, above which changes were considered too significant for accurate normalization. In addition, systematic variation is assessed by calculating the variance (V-value) to determine the optimal number of candidate reference genes required to validate RT-qPCR normalization. If the variance between successive normalization factors (Vn/Vn + 1) is ≤ 0.15, no additional internal controls above n + 1 are required for that particular experimental condition [14,26].

**Figure 5:**
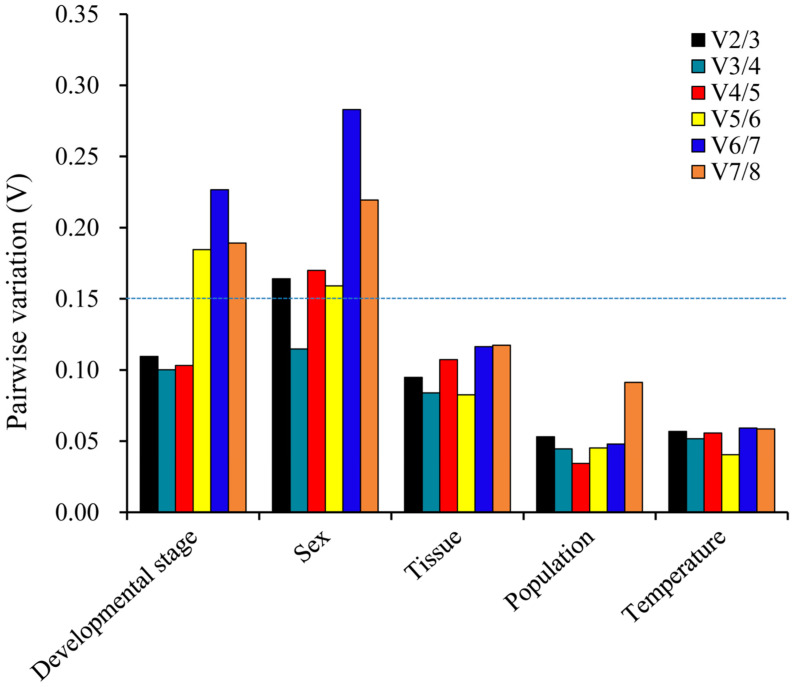
Pairwise variance (V) values were calculated using *geNorm* for five different comparisons: developmental stage, sex, tissue, population and temperature. Values of Vn/Vn+1 ≤ 0.15 indicate that the inclusion of n reference genes in RT-qPCR analyses is sufficient for optimal normalization.

geNorm: The pairwise variants were calculated using *geNorm* in order to determine the minimum number of internal reference genes required as a result of optimal normalization. Our data indicated that each initial V (variation) value (V2/3) was less than 0.15, except for sex, which suggests that optimal normalization can be achieved with two reference genes under the various experimental conditions discussed in this study. For sex, the initial V (variation) value (V3/4) < 0.15 required three reference genes for optimal normalization (Figure 5). From the analysis of Table 2, we determined that *V-ATP-A* and *RPS18* are the best genes for developmental stages. For sex comparison, *α-tubulin*, *RPS18* and *RPL13* emerged as the top choices. In different tissues, *α-tubulin* and *EF1A* were identified as the most suitable genes. Additionally, *RPL13* and *V-ATP-A* exhibited the best gene stability when comparing two different populations. At different temperature experimental settings, *α-tubulin* and *RPS18* were the most suitable genes.

NormFinder: *NormFinder* analysis tells. *V-ATP-A* was the most stably expressed of the eight genes in the developmental stage and sex comparison analysis. *EF1A* showed the greatest stability in different tissue comparisons. For the two different *D. citri* populations, *ArgK* was considered the best gene. In addition, *RPL13* was the most stably expressed gene in samples exposed to different temperatures.

BestKeeper: *BestKeeper* uses the geometric mean, standard deviation (SD) and stability value (SV) of the Ct values of the candidate reference genes in its assessment; lower index scores indicate more stable reference genes. The candidate reference gene was determined to be the most stable gene exhibiting the lowest standard deviation (SD); genes with SD values < 1 were considered acceptable. The analysis of this data set showed that, when comparing developmental stages, *Actin* and *ArgK* all had SD values > 1 and therefore could not be selected as reference genes. Similarly, *Actin*, *ArgK*, *RPL13* and *EF1A* all had SD values > 1, and therefore, they were not suitable as reference genes for comparisons across sexes. However, *18S* was designated as the best gene at developmental stage (0.29) and under population (0.1) comparison conditions, and *V-ATP-A* (0.06) was the most stably expressed gene when analyzing the different sexes. *ArgK* (0.18) and *RPS18* (0.08) were the most stably expressed genes in different tissues and under different temperature comparisons of *D. citri*, respectively.

ΔCt method: We used the Δ*Ct* method to compare “pairs of genes”. Ranking order was determined based on the average Δ*Ct* value; the lower the average SD, the more stable the reference gene. This method revealed that *18S* exhibited the highest stability under different sex and temperature experimental conditions, while *EF1A*, *V-ATP-A* and *Actin* were the most stably expressed genes under different tissue, developmental stages and population conditions, respectively.

### 3.4. Stability Ranking of The Eight Reference Genes

**Figure 6:**
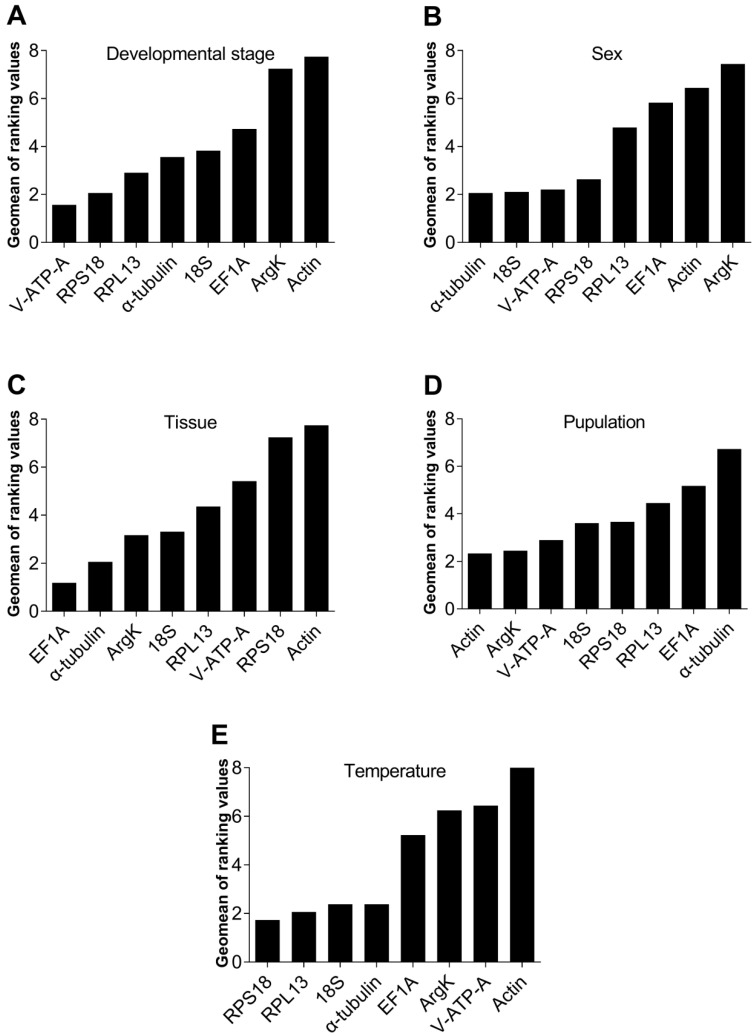
*RefFinder* analyzed the expression stability of the eight candidate internal reference genes of *Dialeurodes citri* under different experimental settings. The lower the calculated Geomean value, using the expression formula provided by the *RefFinder* software, indicates a more stable expression level of the candidate reference gene.

*RefFinder* is a comprehensive evaluation program. It integrates tools from all four of the abovementioned software (*geNorm*, *NormFinder*, *BestKeeper* and Δ*Ct* method) to rank candidate reference genes according to their stability. Based on the findings of *RefFinder*, the integrated reference genes for the developmental stage exhibited varying degrees of stability, with *V-ATP-A*, *RPS18*, *RPL13*, *α-tubulin*, *18S*, *EF1A*, *ArgK* and *Actin* being ranked in descending order of stability (Figure 6A). Similarly, the combined rankings for sex were *α-tubulin*, *18S*, *V-ATP-A*, *RPS18*, *RPL13*, *EF1A*, *Actin* and *ArgK* (Figure 6B), whereas the combined rankings for different tissues were *EF1A*, *α-tubulin*, *ArgK*, *18S*, *RPL13*, *V-ATP-A*, *RPS18* and *Actin* (Figure 6C). Comparing the two sampled populations, we see that *Actin* and *ArgK* had the most stable gene expression levels (Figure 6D); however, *RPS18* and *RPL13* were the most stable under different temperature settings (Figure 6E).

### 3.5. Validation of the Selected Reference Genes

**Figure 7:**
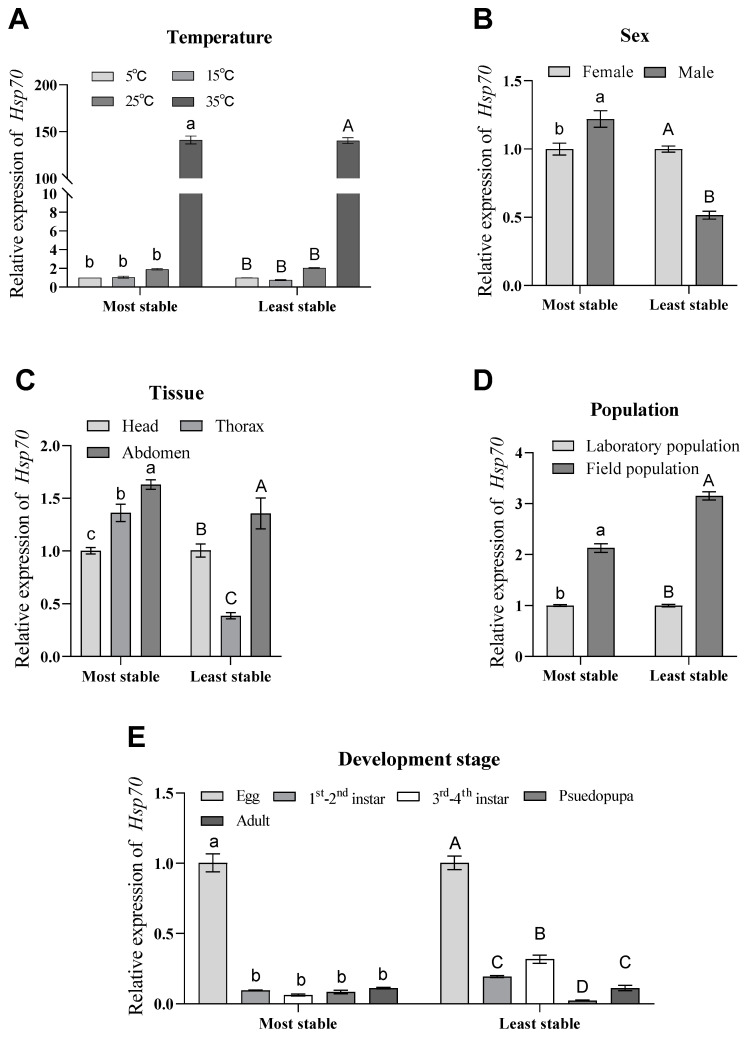
Relative expression of *Hsp70* under different experimental conditions. (**A**) The relative expression of *Hsp70* under different temperature treatment conditions was normalized by selecting the most stable (*RPS18* and *RPL13*) and least stable (*V-ATP-A* and *Actin*) reference genes. (**B**) For sex comparisons, the relative expression of *Hsp70* was calculated using the best reference genes (*α-tubulin*, *18S* and *V-ATP-A*) and the worst reference genes (*EF1A*, *Actin* and *ArgK*) and normalized. (**C**) The relative expression of *Hsp70* in different tissues was normalized to the expression of the optimal genes (*EF1A* and *α-tubulin*) and the worst genes (*RPS18* and *Actin*), respectively. (**D**) Relative expression of *Hsp70* was normalized to the relative expression of the optimal genes (*Actin* and *ArgK*) and the worst genes (*α-tubulin* and *EF1A*) under different population conditions. (**E**) Relative expression of *Hsp70* at different developmental stages was best normalized to the expression of the optimal reference genes (*V-ATP-A* and *RPS18*) and the worst reference genes (*ArgK* and *Actin*). Values are means ± standard error. Different lowercase letters indicate significant differences from the most stable gene (*p* < 0.05). Different uppercase letters indicate significant differences when normalized to the least stable (*p* < 0.05).

To accurately assess and compare the stability of the eight candidate reference genes we selected, we used the *Hsp70* gene, which is stably expressed in all developmental stages of insects. We used the relative expression of *Hsp70* to verify the stability and applicability of the eight candidate reference genes under the five different experimental conditions set. Based on the analysis of the results, we learned that the expression patterns and expression amounts of *Hsp70* were inconsistent under different temperature settings (Figure 7A), different sexes (Figure 7B), different tissues (Figure 7C) and different developmental stages (Figure 7E) of the experimental setups, as found by comparing the most stable candidate reference genes with the least stable candidate reference genes (except for different populations (Figure 7D)) (Figure 7).

## 4. Discussion

In our current study, the stability of eight candidate genes was systematically evaluated under different experimental conditions, among which, *18S* ribosomal RNA, one part of ribosomal RNA [32,51], was stably expressed in most of the studies and under the vast majority of the conditions; consequently, the expression level of this gene was used as a reference [9,22,23,52]. Studies have shown that 18S is the best reference gene for *Sesamia inferens* at different developmental stages and for sex [52]. *Actin* (β-Actin) is a major structural protein which is expressed in many different types of cells at different levels of expression and has been widely used in RT-qPCR analysis [18,20,22,23,24,28,31]. Arginine kinase is ubiquitous in lower and higher invertebrates and is the major phosphokinase in these organisms. It has been used as a reference gene in various research endeavors [18,20,23,32]. For example, arginine kinase (AK) was used as the best reference gene in the *Spodoptera litura* internal reference gene screen [36]. Elongation factor 1 *α* participates in protein synthesis and has commonly served as a benchmark factor in genetic studies involving insects [9,10,23,52]. In *Plutella xylostella*, *EF1A* is the most appropriate reference gene for different insect tissues [37], and it is likewise the most reliable reference gene in different tissues in *Spodoptera exigua* [38]. Ribosomal proteins (RPs) are integral components of ribosomes and are one of the most highly conserved proteins for the evolution of all life [32,52]. The RPL and RPS gene families are frequently utilized as reference genes in the majority of studies; for instance, RP family genes have been utilized in studies of *Sesamia inferens* and *Spodoptera exigua* [19,27,29,38,52]. Tubulin encodes cytoskeletal structural proteins, and the stability of microtubulin varies in the same species under different treatments in many studies [26,27]. *V-ATP-A* is a key component of *Diaphorina citri* (Kuwayama) and is the most stably expressed reference gene during the course of different developmental stages [17,19,24,28].

RT-qPCR technology is extensively used in the detection and quantification of nucleic acids and is one of the major molecular techniques [8,10,16,51]. This is because it enables the end-user to quantify the expression of target genes accurately under many different environments [12,16,51]. The reference genes play a crucial role in the RT-qPCR process, as they are widely recognized as the most stably expressed genes across all cell types [13,17,30,41]. However, the reality is more complex, as there is no “universal” reference gene that can be stably expressed under all experimental conditions [29,30,43]. Furthermore, when researchers use only one reference gene when calculating the expression level of a target gene, it can lead to biased data and errors with interpretation. To precisely ascertain the expression profile of a target gene through RT-qPCR, an appropriate reference gene must be selected [12,27,30]. Although an increasing number of studies have been reported on reference gene screening for Hemiptera, Lepidoptera, Coleoptera, and Diptera insects in recent years [9,13], regrettably, reference gene studies on *D. citri*, an important pest, remain neglected.

A clear finding in our current explorations is that the transcript levels of reference genes are not constant, as they are significantly affected by specific experimental conditions [11,12,16,30]. This observation fits with the findings of many other studies and further confirms the fact that what we have so far considered as “standard” reference genes do not actually have global applicability [12,22,30,33]. More specifically, we found that *V-ATP-A* of *D. citri* showed a very stable and steady expression under experimental conditions, such as developmental stage [17,19], which is the same conclusion as that which other organisms have exhibited, for example, *Coleomegilla maculata* [27]. However, *ArgK* behaved very erratically under certain experimental conditions, and its expression is shown to be unstable in *Tamarixia radiata*; meanwhile, in other organisms, such as *Lysiphlebia japonica* and *Spodoptera litura*, the same gene exhibited relatively stable expression patterns [18,27,36]. This emphasizes the importance of choosing an appropriate reference gene for different organisms and under different experimental environments, and the critical role of this choice in ensuring experimental accuracy [8,12,13,14,15,17,18,19,20,21,25,26,27,31].

Several previous studies have clearly indicated that relying on only one reference gene when performing RT-qPCR analysis may lead to serious bias in normalization [12,18,19,20,21,25,26,27,31], and it is therefore recommended that at least two or more candidate reference genes be selected to ensure the reliability and accuracy of the results [18,19,20,21,25,26,27,30,31]. Indeed, depending on the specific conditions and purpose of the experiment, the necessary number of reference genes required can vary [10,12,16,17,51]. For example, in order to accurately normalize gene expression in *Coleomegilla maculata* at different developmental stages, it may be necessary to refer to five endogenous reference genes, whereas for sex differences, three endogenous reference genes may be sufficient [28,34,44]. Nonetheless, it is important to realize that, in practical studies, opting for an excessive number of reference genes will not only heighten the complexity of the experiment but also increase costs [17,18,20,26,44]. Therefore, in most cases, selecting two rigorously validated reference genes with stable expression may be an ideal strategy to balance practicality and accuracy [19,24,30]. Based on the *geNorm* algorithm, we were able to obtain reliable normalization results for each experimental condition based on two or three stably expressed reference genes. Based on what we learned from the analysis of the *RefFinder* algorithm results, *V-ATP-A* and *RPS18* were found to be the most stable when examining the developmental stages of *D. citri*; *α-tubulin*, *18S* and *V-ATP-A* were the best choices for sex difference studies; for tissue differences, *EF1A* and *α-tubulin* performed the best; for population comparisons, *Actin* and *ArgK* were preferred; and finally, *RPS18* and *RPL13* emerged as the most stable reference genes under different temperature setting conditions. These results further emphasize the variability in reference gene stability observed across organisms, environmental conditions and species.

In our study, *V-ATP-A* showed the highest stability in expression among the reference genes in *D. citri* during different developmental stages [19,35,45]. Since the *V-ATPase* family is closely related to insect growth and development [17,26], and it has been shown that *V-ATP-A* is highly conserved among different insects, it is hypothesized that *V-ATP-A* is the best gene for comparing different growth and developmental stages [35]. However, its high expression levels may mask subtle changes in the expression of target genes. With this in mind, we can utilize other ribosomal machinery genes, such as the RPL and RPS genes, with the RP coding family being considered the best reference genes [33,46,52]. For example, *RPL13* is considered to be the optimal candidate reference gene for studying the different developmental stages of *Tetranychus cinnabarinus* [47]; *RPS18* is the optimal reference gene in developmental stages, tissues, temperatures and host plants of *Henosepilachna vigintioctopunctata* [22]. However, according to our findings, we inferred that *RPS18* is the prime candidate as a reference gene for contrasting different temperature conditions, while *RPL13* is the second most stably expressed reference gene. Moreover, *RPS18* was second only to *V-ATP-A* in terms of stability in comparisons across developmental stages. According to previous research, *ArgK* has been deemed unsuitable as a reference gene [18,26,27]; however, our findings indicate that *ArgK* ranked second as the most stable reference gene under different population settings. In addition, *α-tubulin* is commonly employed as a reliable reference gene under conditions of insect temperature experiments [22], but during our validation, *α-tubulin* showed less stability than the RP genes, whereas *α-tubulin* showed the most stability under sex and tissue comparison conditions. Furthermore, *Actin* was the gene with the most stable expression across populations in our study. Additionally, these results further demonstrate that the stability of reference genes varies across organisms or environmental conditions and across species [24,28,48].

In summary, we examined the expression profiles of eight candidate reference genes in five experimental conditions (developmental stage, sex, tissue, population and temperature), using five commonly used algorithms (*geNorm*, *NormFinder*, *BestKeeper*, Δ*Ct* method, and *RefFinder*) [14,26,31,39,40]. A specific set of reference genes was recommended for each experimental condition. Our analysis of these combined results highlighted the absence of a universal reference gene for all conditions and emphasized the variable response of reference genes to different experimental conditions. This study marks a critical first step towards establishing a standardized RT-qPCR protocol for functional genomics studies of *D. citri*. In order to further explore the role of reference genes in *D. citri*, we accurately assessed the relative gene expression levels of *Hsp70* under different experimental condition setups [7,9]. The findings re-emphasize that the selection of appropriate reference genes is crucial to ensure the stability of the RT-qPCR analysis [12,16,30]. Otherwise, illogical misuse of the reference genes and incorrect expression patterns of the internal reference genes will affect the correct understanding of the target genes [18,49,50].

## 5. Conclusions

In conclusion, we selected eight potential reference genes for the citrus whitefly and evaluated their consistency under five experimental conditions. To the best of our knowledge, this is the first study involving the identification and subsequent comparison of RT-qPCR reference genes for the citrus whitefly under five different experimental conditions. We expect that these findings will simplify future studies on the expression patterns of citrus whitefly target genes and thus provide valuable insights into the functional studies of these target genes.

## Figures and Tables

**Table 1:** Reference genes used in this study.

Gene	Primer Sequences (5′-3′)	Length (bp)	Efficiency (%)	R2	Linear Regression
*18S-F*	GACGGGGTCTCGTTCGTTAT	119	99.86	0.9996	y = −3.3254x + 11.837
*18S-R*	CACCGGAAGGATTGACAGAT				
*V-ATP-A-F*	GTCAGGGTTGCCAAGACACT	118	103.39	0.9965	y = −3.2433x + 23.339
*V-ATP-A-R*	AATTTCAGGACGTTTGGCTG				
*Actin-F*	AGAAGCCCTCTTCCAACCAT	127	100.37	0.9998	y = −3.313x + 19.51
*Actin-R*	GGAGAGAACTGTGTTGGCGT				
*ArgK-F*	GCAACACCTGATTGGATGAC	118	97.49	0.9988	y = −3.3836x + 20.294
*ArgK-R*	TTTAGATGCCATCCAGCCAC				
*α-tubulin-F*	GGAGGAAACAATTTGACCGA	126	94.07	0.9996	y = −3.4727x + 20.03
*α-tubulin-R*	GAACACTCCGACTGTGCCTT				
*EF1A-F*	TTTAGATGCCATCCAGCCAC	120	91.12	0.9991	y = −3.5548x + 17.789
*EF1A-R*	ACACCGGTTTCAACACGAC				
*RPL13-F*	CGTTTGCAGTGATAGCACGA	120	95.09	0.9990	y = −3.4455x + 18.943
*RPL13-R*	AGCTGAAGAAGGGAGAGGCT				
*RPS18-F*	CTCACAGATCACAGCCTCCA	108	91.79	0.9992	y = −3.5357x + 19.349
*RPS18-R*	ACACGGAGTCCCCAATGAT				

**Table 2:** Stability of candidate reference gene expression under four calculation methods (*geNorm*, *NormFinder*, *BestKeeper* and Δ*Ct* method) evaluated in five different experiments, respectively.

Conditions	Rank	geNorm		NormFinder		BestKeeper		ΔCt	
		Gene	Stability	Gene	Stability	Gene	Stability	Gene	Stability
Developmental stage	1	*V-ATP-A*	0.315	*V-ATP-A*	0.158	*18S*	0.29	*V-ATP-A*	0.82
	2	*RPS18*	0.315	*RPL13*	0.306	*α-tubulin*	0.66	*RPS18*	0.84
	3	*RPL13*	0.348	*RPS18*	0.362	*RPS18*	0.66	*RPL13*	0.87
	4	*EF1A*	0.398	*α-tubulin*	0.567	*RPL13*	0.82	*α-tubulin*	0.96
	5	*α-tubulin*	0.464	*EF1A*	0.697	*EF1A*	0.82	*EF1A*	0.99
	6	*18S*	0.689	*18S*	1.124	*V-ATP-A*	0.87	*18S*	1.36
	7	*ArgK*	0.965	*ArgK*	1.41	*Actin*	1.61	*ArgK*	1.61
	8	*Actin*	1.139	*Actin*	1.484	*ArgK*	1.69	*Actin*	1.66
Sex	1	*α-tubulin*	0.131	*V-ATP-A*	0.145	*V-ATP-A*	0.06	*18S*	0.95
	2	*RPS18*	0.131	*18S*	0.145	*18S*	0.22	*α-tubulin*	0.96
	3	*RPL13*	0.374	*α-tubulin*	0.401	*α-tubulin*	0.7	*RPS18*	0.99
	4	*EF1A*	0.423	*RPS18*	0.569	*RPS18*	0.79	*V-ATP-A*	1.03
	5	*18S*	0.594	*RPL13*	1.124	*Actin*	1.12	*RPL13*	1.24
	6	*V-ATP-A*	0.716	*EF1A*	1.277	*ArgK*	1.17	*EF1A*	1.34
	7	*Actin*	1.078	*Actin*	1.654	*RPL13*	1.18	*Actin*	1.72
	8	*ArgK*	1.251	*ArgK*	1.721	*EF1A*	1.29	*ArgK*	1.77
Tissue	1	*α-tubulin*	0.175	*EF1A*	0.225	*ArgK*	0.18	*EF1A*	0.53
	2	*EF1A*	0.175	*18S*	0.236	*EF1A*	0.21	*α-tubulin*	0.54
	3	*RPL13*	0.256	*α-tubulin*	0.296	*α-tubulin*	0.25	*18S*	0.56
	4	*18S*	0.309	*ArgK*	0.346	*V-ATP-A*	0.31	*RPL13*	0.59
	5	*ArgK*	0.41	*RPL13*	0.38	*18S*	0.36	*ArgK*	0.61
	6	*V-ATP-A*	0.453	*V-ATP-A*	0.554	*RPL13*	0.42	*V-ATP-A*	0.7
	7	*RPS18*	0.564	*RPS18*	0.764	*Actin*	0.72	*RPS18*	0.87
	8	*Actin*	0.676	*Actin*	0.924	*RPS18*	0.73	*Actin*	1.01
Population	1	*RPL13*	0.118	*ArgK*	0.077	*18S*	0.1	*Actin*	0.27
	2	*V-ATP-A*	0.118	*Actin*	0.084	*ArgK*	0.15	*V-ATP-A*	0.3
	3	*RPS18*	0.149	*RPS18*	0.189	*Actin*	0.27	*ArgK*	0.31
	4	*EF1A*	0.172	*18S*	0.193	*α-tubulin*	0.36	*RPS18*	0.31
	5	*Actin*	0.183	*V-ATP-A*	0.202	*RPS18*	0.37	*EF1A*	0.33
	6	*ArgK*	0.218	*EF1A*	0.233	*EF1A*	0.4	*18S*	0.36
	7	*18S*	0.255	*RPL13*	0.334	*V-ATP-A*	0.41	*RPL13*	0.37
	8	*α-tubulin*	0.375	*α-tubulin*	0.727	*RPL13*	0.5	*α-tubulin*	0.74
Temperature	1	*α-tubulin*	0.115	*RPL13*	0.114	*RPS18*	0.08	*18S*	0.29
	2	*RPS18*	0.115	*18S*	0.116	*α-tubulin*	0.08	*RPL13*	0.29
	3	*RPL13*	0.158	*RPS18*	0.174	*RPL13*	0.12	*RPS18*	0.3
	4	*18S*	0.192	*α-tubulin*	0.184	*18S*	0.19	*α-tubulin*	0.3
	5	*EF1A*	0.235	*EF1A*	0.194	*V-ATP-A*	0.23	*EF1A*	0.32
	6	*ArgK*	0.248	*ArgK*	0.208	*EF1A*	0.3	*ArgK*	0.32
	7	*V-ATP-A*	0.301	*V-ATP-A*	0.444	*ArgK*	0.34	*V-ATP-A*	0.48
	8	*Actin*	0.349	*Actin*	0.461	*Actin*	0.48	*Actin*	0.49

## Data Availability

All the data is in this manuscript.
